# Older adults can improve compensatory stepping with repeated postural perturbations

**DOI:** 10.3389/fnagi.2015.00201

**Published:** 2015-10-21

**Authors:** Bauke W. Dijkstra, Fay B. Horak, Yvo P. T. Kamsma, Daniel S. Peterson

**Affiliations:** ^1^Department of Human Movement Sciences, University Medical Centre Groningen, University of GroningenGroningen, Netherlands; ^2^Veterans Affairs Portland Health Care CentrePortland, OR, USA; ^3^Balance Disorders Laboratory, Department of Neurology, Oregon Health and Science UniversityPortland, OR, USA

**Keywords:** postural motor learning, compensatory stepping, aging, balance, posture, rehabilitation

## Abstract

The ability to respond quickly and accurately to an external perturbation with a stepping response is critical to avoid falls and this ability is impaired in older, compared to young adults. However, little is known about whether young and older adults improve compensatory stepping responses similarly with practice. This study compares the extent to which young and older adults can improve, retain, and generalize postural compensatory steps in response to external perturbations. Centre of mass displacement, step characteristics and lower leg muscle activation latencies were measured during one training session of compensatory stepping in response to large surface translations in 13 young and 12 older adults. Retention was tested 24 h later. Older adults decreased their center of mass displacements over repeated exposure to large surface translations in both the anterior and posterior directions and retained these improvements. In contrast, young adults only showed adaptation and retention of forward stepping responses. Neither group was able to generalize improvements in stepping responses across directions. These results suggest step training may be beneficial for older adults, however additional, multidirectional training may be necessary to facilitate generalization of postural stepping responses for any direction of a slip or trip.

## Introduction

Each year, 30–60% of healthy older adults fall and 10–20% of these falls result in injury, hospitalization, and/or death (Rubenstein, 2006). Falls also lead to a loss of independence, decline in health status and decreased quality of life (Roe et al., 2009), and fall-related injuries are associated with substantial economic costs (Stevens et al., 2006). A common contributor to falls is failure to recover from an external perturbation such as a push or a slip (Robinovitch et al., 2013). The ability to make compensatory postural responses (i.e., stepping or arm movements) to recover equilibrium in response to external perturbations is critical to avoid falls (Maki and McIlroy, 2006).

Older adults exhibit less effective postural responses to external perturbations than young adults (Horak et al., 1989). Older adults step to smaller perturbations than young (Jensen et al., 2001; Mille et al., 2003), and more often take multiple compensatory steps (McIlroy and Maki, 1996; Maki et al., 2000; Mille et al., 2005, 2013) in response to both anteroposterior and lateral perturbations. Further, stepping strategies for lateral stepping can be altered in older adults, as young adults usually use a single sideways step, whereas older adults often cross one foot over the other with multiple steps which may lead to foot collisions, missteps, and falls (Maki et al., 2000; Mille et al., 2005).

Given the inefficient compensatory stepping in older adults, and the importance of compensatory stepping for fall prevention (Carty et al., 2015), it is important to determine whether automatic postural stepping responses can be improved with training. To our knowledge, only one study has looked at this question for older adults. Mansfield et al. (2010) showed that older adults improve compensatory stepping through compensatory step training. In this report, older adults decreased the frequency of multiple stepping responses and foot collisions after 18 sessions over 6 weeks of practice. While this study provided insight into the ability of older adults to improve compensatory stepping with extended training, only pre- and post-training measurements were taken, and training was restricted to older adults, providing little insight into the learning process during the intervention or age-related differences in learning.

A clear understanding of the effect of age on postural motor learning is also critical for balance rehabilitation. To date, little research has investigated the effects of age on postural motor learning, and fewer still have investigated learning of reactive balance tasks (Pavol et al., 2002, 2004; Van Ooteghem et al., 2009, 2010). Two studies show that young and older adults both decrease fall incidence at a similar rate over repeated exposure to forward surface translations during a sit-to-stand task (Pavol et al., 2002, 2004). The decrease in fall incidence is achieved through both proactive adaptations of the sit-to-stand task performance and adaptive changes in the reactive response to slipping. Since the surface translations were always in the same direction in these studies, it is unclear how older adults improve postural responses when the perturbation direction is unknown (Pavol et al., 2002, 2004). The ability to improve the quality of stepping responses to unexpected direction of perturbations is particularly important given that the direction and size of environmental perturbations are typically unknown. Van Ooteghem et al. (2009, 2010) compared postural motor learning of feet-in-place postural responses in young and older adults during a continuous perturbation task, in which the support surface moved slowly and continuously forward and backward. Both young and older adults improved their postural motor control over repeated exposures, and improvements were retained 24 h later. The work of Van Ooteghem et al. (2009, 2010) suggests older adults retain their capacity for postural motor learning during stance, but does not address the question- can compensatory stepping [a more complex and cortically controlled task (Jacobs and Horak, 2007)] be similarly learned by young and older adults?

The purpose of this study is to determine whether young and older adults exhibit similar improvements in compensatory stepping over repeated exposure to external perturbations and if improvements are similarly retained overnight and generalized to another direction. Based on previous studies on postural motor learning (Pavol et al., 2002, 2004; Van Ooteghem et al., 2009, 2010), we hypothesized that despite performance deficits, older adults will be able to adapt and retain their compensatory stepping in response to repeated external perturbations similar to young adults.

## Methods

### Participant characteristics

Participant characteristics are described in Table 1. Before inclusion in this study, young and older adults were screened to ensure that they did not have any medical condition affecting postural control or stepping responses. One young adult was excluded from the analysis. This individual interpreted the instructions differently than the other participants, resulting in center of mass (CoM) displacements >2 standard deviations from the group mean. The Institutional Review Board of the Oregon Health and Science University approved the methods used in this study. All participants provided consent prior to participation in the study.

**Table 1:** **Participant characteristics**.

	**Old adults (*n* = 12)**	**Young adults (*n* = 13)**	***p*-value**
Sex (n females)	6	6	
Age (y)	68 ± 7	28 ± 4	< 0.001
Weight (kg)	75 ± 9	68 ± 2	0.11
Height (cm)	166 ± 34	174 ± 6	0.02
Leg length (cm)	89 ± 9	91 ± 4	0.34

*Values are mean ± SD*.

### Experimental protocol

Participants visited the laboratory on two consecutive days with approximately 24 h between visits. The participants stood on a moveable platform with arms folded across the chest to minimize compensatory arm responses and were instructed to distribute their weight equally over both feet. Perturbations were delivered via surface translations, a reliable manner to examine compensatory stepping responses (Crenshaw and Kaufman, 2014). Participants were instructed “not to anticipate upcoming perturbations and to react naturally to the perturbation when trying to keep balance.” Open-ended instructions were given to avoid altering the natural, compensatory stepping responses. Participants wore a harness attached to the ceiling to protect against falls. The harness provided no body weight support.

To familiarize subjects with the perturbations and eliminate excessive fear and/or startle responses, both days started with three “first perturbations,” which consisted of a forward (9 cm, 18 cm/s) and left (9 cm, 14.6 cm/s) translation and a toes-up rotation (4°, 20°/s). To determine the size of perturbation for each subject to elicit a natural, automatic stepping response but not a fall, the step threshold was determined for all directions (forward, backward, left, right) for each participant. The determination of the step threshold consisted of 3 perturbations per direction, starting with a small perturbation (forward and backward: 9 cm, 18 cm/s; left and right: 9 cm, 14 cm/s) and increased to a large perturbation (forward and backward: 15 cm, 56 cm/s; left and right: 15 cm, 21 cm/s). Subsequently, as a baseline measure for generalization, participants underwent 5 leftward and 5 rightward translations in random order. The directions of all perturbations were randomly ordered to eliminate anticipatory biomechanical changes (e.g., leaning, change in muscular tone, etc.). The protocol continued with the postural motor training consisting of 25 forward and 25 backward translations in random order (administered in 5 blocks of 10 perturbations). On Day 2, participants were exposed to exactly the same initial (3 perturbations), same determination of the step threshold (12 perturbations), and generalization trials (10 left/right perturbations). Then, the retention test took place, consisting of 5 forward and 5 backward translations (random order). Short (< 5 min) rest periods of sitting were allowed between blocks. The experimental protocol is summarized in Table 2.

**Table 2:** **Protocol**.

		**Number of perturbations per platform translation direction**			
		**Forward**	**Backward**	**Left**	**Right**
Day 1	^*^First perturbations	1		1	
	Determination of step threshold	3	3	3	3
	^‡^Baseline lateral stepping			5	5
	^‡^Postural motor training	25	25		
Day 2	^*^First perturbations	1		1	
	Determination of step threshold	3	3	3	3
	^‡^Generalization lateral stepping			5	5
	^‡^Retention postural motor training	5	5		

* *One toes up rotation was also included in the first perturbations, data not reported*.

‡ *Perturbation sequence was randomly ordered across directions*.

### Data analysis

The maximum displacement of the whole-body CoM was taken as the primary measure to characterize the global postural control performance in response to perturbations. CoM for each segment was calculated using segment kinematics and anthropometric data from 36 reflective markers placed on anatomic landmarks (Chandler et al., 1975; Vaughan et al., 1992). A Motion Analysis system (Motion Analysis Corporation, Santa Rosa, California) sampling at 120 Hz provided three-dimensional spatial coordinates of the markers. Marker data were filtered with a 4th order Butterworth low pass filter with a frequency of 5 Hz for CoM calculations.

Step characteristics included first step latency (onset of surface translation to onset of foot off the force plate), first step length (distance between the stance foot and the swing foot at first foot contact) and number of steps (steps until maximum anterior or posterior CoM displacement). All variables were calculated via a customized, semi-automated program written in Matlab®. Identification of lateral step strategy (i.e. side-step, cross-over, etc.) is discussed in the Supplementary Material.

Muscle response latencies were determined via electromyography (EMG). Surface electrodes were placed bilaterally on the tibialis anterior and the medial gastrocnemius muscles. EMG signals were amplified at a gain of 5000–10,000, band-pass filtered from 75 to 470 Hz, and full-wave rectified. A linear envelope was created by low-pass filtering at 100 Hz. The latency of each muscle burst was identified as the first sustained activity lasting at least 25 ms, and greater than two standard deviations above the baseline, using an interactive graphing function programmed in MATLAB. The collection of EMG data of one older adult failed due to technical problems.

### Statistical analysis

Statistical analyses were performed with SPSS version 20. Backward and forward steps were analyzed separately. To assess improvements due to adaptation within the Day 1 training session, the motor training was divided into 5 blocks of five trials for backward compensatory stepping and 5 blocks of five trials for forward compensatory stepping. Similarly, forward/backward retention stepping (Day 2) was assessed as one block of 5 trials for backward and one block of 5 trials for forward stepping. The 5 right and 5 left translations to evaluate generalization effects were considered as one block. The assumption of normality was checked with the Shapiro–Wilk test. If data were not normally distributed, a log transformation was performed on the data. If, after the log transformation, the assumption of normality was still violated, a non-parametric test was used. A two-tailed, independent *t*-test or Mann–Whitney test was used to determine if young and older adults differed in Block 1 (baseline).

A mixed ANOVA, with *group* as between subjects factor and *block* as within subject factor, determined training related adaptations and the *group by block* interaction effect for variables that were normally distributed. For non-normally distributed data, two separate Friedman tests for both groups determined training-related adaptations. A Greenhouse–Geisser correction was applied if the assumption of sphericity was violated. If a *group by time* interaction was found, a paired *t*-test or Wilcoxon signed-rank test was used to determine if either group adapted between Block 1 and Block 5 on Day 1. The retention of improvement was determined with a paired samples *t*-test or Wilcoxon signed-rank test in which Block 1 on Day 1 was compared to the same block on Day 2. Similarly, generalization of the postural motor training to lateral translations were determined with a paired samples *t*-test or Wilcoxon signed-rank test comparing translations on Day 1 with those on Day 2. For the CoM displacement in backward and forward compensatory stepping the Pearson correlation coefficient between the initial performance (Block 1) and the change in performance over training (Block 1–Block 5) was calculated. Alpha was set at *p* = 0.05.

## Results

All participants independently recovered balance with compensatory steps at the “large” perturbation size (15 cm, 56 cm/s) and were able to complete the motor training program. Three young adults recovered balance in response to the backward surface translations without taking a compensatory forward step in 2.7% of trials. Means, standard deviations, and statistical outputs for all variables are shown in Table 3.

**Table 3:** **Adaptations during the motor training (represented as mean ± SD)**.

		**Block 1**	**Block 5**	**Day 2**	**Mixed model ANOVA**		
					**Group effect**	**Time effect**	**Group × time interaction effect**
**BACKWARD COMPENSATORY STEPPING**							
CoM displacement (m)	HO	0.31±0.04	0.25±0.02	0.27±0.03	*F*_(1, 23)_ = 0.29	***F*_(2.35, 54.04)_ = 6.10**	***F*_(2.35, 54.04)_ = 3.79**
	HY	0.27±0.05	0.27±0.06	0.27±0.04	*p* = 0.60	***p* ≤ 0.01**	***p* ≤ 0.05**
Number of steps^*^	HO	1.98±0.54	1.28±0.37	1.58±0.45		^*^χ^2^(4) = 18.66, *p* ≤ 0.001	
	HY	1.25±0.37	1.15±0.32	1.08±0.10		^*^χ^2^(4) = 5.27, *p* = 0.27	
Step length (m)	HO	0.25±0.11	0.27±0.08	0.25±0.08	*F*_(1, 23)_ = 3.79	*F*_(1.76, 40.57)_ = 0.32	*F*_(1.76, 40.57)_ = 1.29
	HY	0.31±0.06	0.30±0.06	0.33±0.04	*p* = 0.06	*p* = 0.70	*p* = 0.28
Step latency (s)	HO	0.26±0.05	0.25±0.02	0.26±0.03	*F*_(1, 23)_ = 0.002	*F*_(2.09, 48.05)_ = 0.74	*F*_(2.09, 48.05)_ = 2.09
	HY	0.25±0.04	0.27±0.05	0.25±0.03	*p* = 0.96	*p* = 0.49	*p* = 0.13
Tibialis anterior activation latency (ms)	HO	123±11	120±9	125±11	*F*_(1, 22)_ = 3.11	*F*_(4, 88)_ = 2.15	*F*_(4, 88)_ = 1.30
	HY	115±9	114±9	114±9	*p* = 0.09	*p* = 0.08	*p* = 0.28
**FORWARD COMPENSATORY STEPPING**							
CoM displacement (m)	HO	0.29±0.11	0.24±0.06	0.25±0.05	*F*_(1, 23)_ = 0.67	***F*_(2.26, 51.91)_ = 9.17**	*F*_(2.26, 51.91)_ = 0.77 *p* = 0.48
	HY	0.26±0.04	0.24±0.06	0.23±0.03	*p* = 0.42	***p* ≤ 0.001**	
Number of steps^*^	HO	1.47±0.45	1.08±0.29	1.10±0.25		^*^χ^2^(4) = 20.06, *p* ≤ 0.001	
	HY	1.14±0.28	1.02±0.06	1.03±0.08		^*^χ^2^(4) = 6.38, *p* = 0.18	
Step length (m)	HO	0.30±0.11	0.28±0.09	30±0.08	*F*_(1, 23)_ = 0.01	***F*_(2.00, 46.19)_ = 5.83**	*F*_(2.00, 46.19)_ = 1.98
	HY	0.32±0.04	0.27±0.05	0.27±0.06	*p* = 0.76	***p* ≤ 0.01**	*p* = 0.15
Step latency (s)^*^	HO	0.35±0.12	0.34±0.10	0.32±0.04		^*^χ^2^(4) = 5.48, *p* = 0.24	
	HY	0.36±0.13	0.40±0.13	0.42±0.18		^*^χ^2^(4) = 15.09, *p* ≤ 0.01	
Gastrocnemius activation latency (ms)	HO	133±23	125±15	126±14	*F*_(1, 22)_ = 0.59	*F*_(4, 88)_ = 1.99	*F*_(4, 88)_ = 0.25
	HY	126±10	123±12	122±11	*p* = 0.45	*p* = 0.10	*p* = 0.73

*HO, healthy older adults; HY, healthy young adults. Bold text indicates statistical significance (p < 0.05)*.

* *Friedman's test of repeated measures across time (non-parametric)*.

### COM displacement

#### Backward stepping

**Figure 1:**
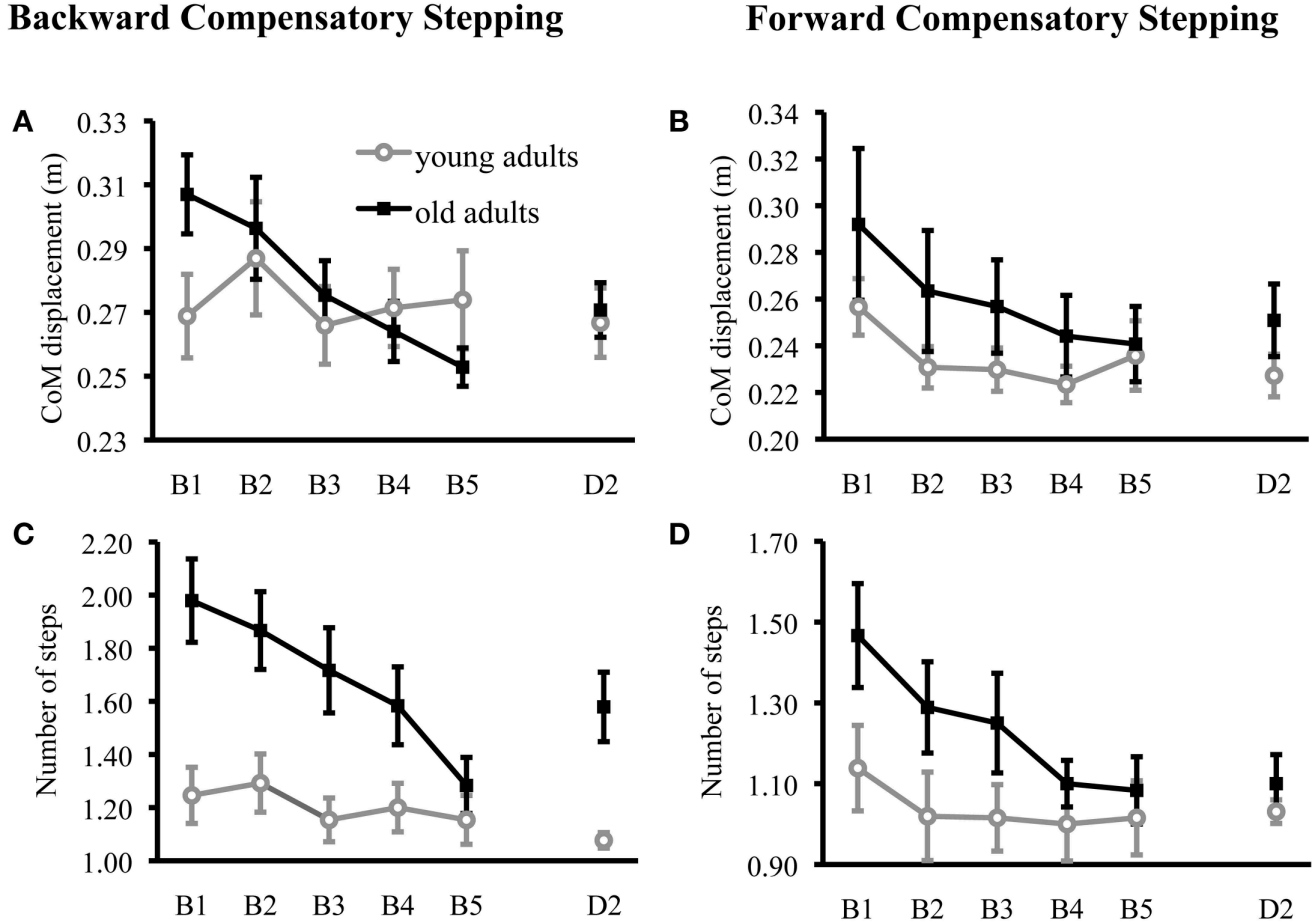
**Forward and backward compensatory step performance**. Center of mass displacement (CoM) in the anteroposterior direction for healthy young and healthy older adults during compensatory backward **(A)** and forward **(B)** stepping are displayed for the motor training (block 1: B1–block 2: B2) and the retention test on day 2 (D2). Number of steps are displayed for young and older adults during compensatory backward **(C)** and forward **(D)** stepping are displayed for the motor training and the retention test on day 2. Values are represented as group mean with SE.

CoM displacement was significantly larger in older adults compared to young adults at baseline; *t*_(23)_ = 2.11, *p* = 0.05. Surprisingly, CoM displacement was significantly reduced over training in the older, but not younger, adults (Table 3; Figure 1A). Specifically, older adults significantly reduced CoM displacement from Block 1 to Block 5 on Day 1 [*t*_(11)_ = 3.93, *p* = 0.01], whereas young adults did not [*t*_(12)_ = −0.05, *p* = 0.96]. Older adults also retained improvements on Day 2 [*t*_(11)_ = 2.51, *p* < 0.05]. Older adults showed a significant relationship between initial performance and change in performance over training such that those with the largest CoM displacements reduced their CoM displacements the most (*r* = 0.88, *p* < 0.001), whereas young adults did not (*r* = 0.32, *p* = 0.30).

#### Forward stepping

CoM displacement did not significantly differ between young and older adults at baseline [*t*_(23)_ = 0.74, *p* = 0.47] and decreased over training in both groups (Table 3; Figure 1B). Young adults retained improvements in compensatory stepping on Day 2 [*t*_(12)_ = 2.41, *p* = 0.05], whereas older adults showed a trend for retention [*t*_(11)_ = 2.08, *p* = 0.06]. Older adults showed a significant relationship between initial performance and improvement in performance over training (*r* = 0.95, *p* < 0.001), where young adults did not (*r* = 0.47, *p* = 0.11).

#### Lateral stepping

**Figure 2:**
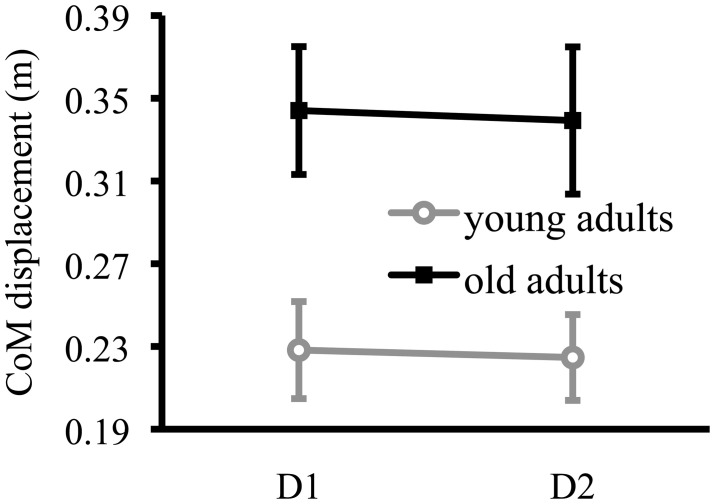
**Lateral compensatory stepping performance**. Center of mass displacement (CoM) in the lateral direction for healthy young and healthy older adults during lateral compensatory stepping at baseline (D1) and retention (D2). Values are represented as group mean with SE.

The reduced CoM displacement during training in backward and forward compensatory stepping did not generalize to lateral compensatory stepping. Body CoM displacements during lateral compensatory stepping was not significantly different at Day 2 compared to Day 1 for young [*t*_(12)_ = 0.26, *p* = 0.78] or older adults [*t*_(11)_ = 0.23, *p* = 0.83] (Figure 2). Body CoM displacement during lateral compensatory stepping was significantly larger in older adults compared to young adults at baseline; *t*_(23)_ = 3.01, *p* = 0.01. The results for lateral stepping strategies are displayed in the Supplementary Material.

### Number of steps

#### Backward stepping

Older adults took significantly more steps at baseline than young adults (*U* = 27, *p* = 0.01), and older adults were able to significantly reduce their number of steps over training (Table 3; Figure 1C). Similar to lack of training effect in young adults on CoM displacements during backward stepping, young adults did not reduce their number of backward steps across training (Table 3). Reduction in the number of steps with training was retained by older adults (*z* = −2.53, *p* = 0.01).

#### Forward stepping

Older adults took significantly more steps at baseline than young adults (*U* = 42, *p* = 0.05), and older adults were able to significantly reduce their number of steps over training (Table 3; Figure 1D). Number of steps in forward compensatory stepping did not change across training in young adults (Table 3). Reduction in the number of steps over training was retained by older adults (*z* = −2.54, *p* = 0.01).

### Step length

#### Backward stepping

Young adults took significantly longer steps than older adults (*U* = 28, *p* = 0.01). However, step length did not change over training for young or older adults (Table 3).

#### Forward stepping

Step length did not differ between young and older adults at baseline; *t*_(14.312)_ = −0.58, *p* = 0.57. Young and older adults both decreased step length over training (Table 3), and young adults, but not older adults, retained these adaptations [young adults: *t*_(12)_ = 2.45, *p* = 0.05; older adults: *t*_(11)_ = −0.19, *p* = 0.86].

### Step latency

#### Backward stepping

Step latency did not differ between young and older adults [*t*_(23)_ = 0.81, *p* = 0.42] and no changes were observed over training (Table 3).

#### Forward stepping

Step latency did not differ at baseline between young and older adults (*U* = 75.59, *p* = 0.89). Forward step latency did not change over training for older adults (Table 3), whereas young adults demonstrated a subtle, but significant, increase in step latency over training (Table 3). Young adults showed retention of adaptation on Day 2; *z* = −2.06, *p* = 0.05.

### Muscle activation latency

#### Backward stepping

Both young and older adults showed a trend for reduced tibialis anterior latencies over backward step training (Table 3). Tibialis anterior activation latency showed a trend for a difference between young and older adults at baseline [*t*_(22)_ = 1.78, *p* = 0.09]. Adaptations were not retained on Day 2 for either young adults [*t*_(12)_ = −1.69, *p* = 0.12] or older adults [*t*_(10)_ = −1.49, *p* = 0.17].

#### Forward stepping

Gastrocnemius activation latency did not significantly differ between young and older adults at baseline; [*t*_(22)_ = 1.07, *p* = 0.30]. Both young and older adults showed a trend for decreased gastrocnemius activation latencies over forward stepping training (Table 3), but these adaptations were not retained on Day 2 for young adults [*t*_(12)_ = 1.36, *p* = 0.20] or older adults [*t*_(10)_ = 1.20, *p* = 0.26].

## Discussion

To our knowledge, this is the first study to investigate the effect of age on the postural motor learning process in compensatory stepping for stance equilibrium. Our results agree with previous research (McIlroy and Maki, 1996; Maki et al., 2000; Jensen et al., 2001; Mille et al., 2003, 2005, 2013) demonstrating that older adults exhibit poorer compensatory stepping compared to young adults. We extend current knowledge by showing that older adults are able to improve their compensatory stepping responses similarly, or even more than, young adults. However, the lack of improvement in backward compensatory stepping in young adults may be related to a floor effect, as further improvement in this group may have been impossible or unnecessary. The improvement with practice in older adults was observed for both forward and backward compensatory stepping, and given the random presentations of forward and backward perturbations, was not due to anticipating the direction of perturbations. Furthermore, older adults retained these improvements over 24 h, a sign of postural motor learning. Improvements made in compensatory backward and forward stepping did not generalize to lateral compensatory stepping for either group. Given the importance of compensatory stepping in avoiding falls, understanding how compensatory stepping is degraded in older adults, and the degree to which it can be improved via training is important for the development of rehabilitation programs targeting fall prevention.

Young and older adults improved compensatory stepping at a similar rate during forward stepping. Interestingly, during backward stepping, older adults exhibited improved performance over time, whereas young adults did not. At baseline, almost all young adults responded with one backward step to the external perturbation, indicating a close to optimal performance and leaving less room for improvement. In contrast, older adults typically responded with multiple steps, allowing room for improvement in performance. Indeed, previous research showed that young adults are able to improve backward compensatory stepping performance to repeated perturbations when the size of perturbations are large enough to elicit multiple step (Patel and Bhatt, 2015). Thus, it is likely that the young adults in the current study did not improve backward stepping because they experienced a floor effect. These results suggest that while initial performance to stepping differs across young and older adults, older adults demonstrate a preserved ability to improve compensatory stepping to match performance of young adults.

The effects of age on postural motor learning is not well understood. A recent study suggested that improvements in postural responses with training are mediated by altering central sensitivity to perturbations and may be an optimization strategy for both stability and energetic considerations (Welch and Ting, 2014). Studies that investigated the effects of age on feet-in-place postural responses during a continuous perturbation task (Van Ooteghem et al., 2009, 2010) and a single direction of postural stepping responses during a sit-to-stand task (Pavol et al., 2002, 2004) have shown that older adults can improve performance at a similar rate as young adults. The current study builds on these results because it shows older adults can improve postural compensatory stepping even when the direction of the external perturbation is not known. Ability to adapt compensatory steps, especially when perturbation direction is unknown, is crucial for fall prevention. Importantly, our results also demonstrate that older adults can retain compensatory stepping improvements after 24 h similar to young adults. The relative permanency of changes in movement is central to motor learning (Schmidt and Lee, 2005), and this retention is critical for effective neurorehabilitative practice.

Another important component of motor learning is the ability to generalize what has been learned. It has been shown that aging does not affect generalization of learning in upper limb motor tasks (Seidler, 2007). Furthermore, our laboratory showed that young and older adults can generalize improvements made while standing on a continuously oscillating surface to a similar task with a new perturbation sequence (Van Ooteghem et al., 2009, 2010). However, the degree to which learning of postural reactions generalizes across stepping tasks is not known. The current study did not demonstrate generalization from compensatory stepping in the anteroposterior direction to lateral compensatory stepping for either young or older adults. The fact that young adults did not show generalization may indicate that lateral compensatory stepping is too distinct from anteroposterior compensatory stepping to benefit from generalized learning. Rehabilitation programs targeting fall prevention should, therefore, include lateral external perturbations to target lateral instability, as well as anteroposterior perturbations. Alternatively, training protocols with increased variability of perturbation directions, size and speed may improve generalization of effects (Schmidt and Lee, 2005).

The improvement in postural stepping responses to anteroposterior external perturbations in older adults, measured by reduced body CoM displacement, may have stemmed from several sources. During forward stepping, number of steps, and step length were both reduced across training blocks. During backward stepping however, the decrease in number of steps to recover equilibrium seems to be the major cause of reduction in CoM displacement. EMG onset during backward stepping also showed a subtle reduction in older adults over the course of training; however, no concomitant changes were observed in associated step latencies, suggesting the slightly improved EMG onset times had little functional benefit on CoM displacements.

Several limitations should be noted. First, participants were instructed to not think about the perturbation that was coming and to respond naturally. These instructions may have increased variability in stepping responses between subjects and between trials. However, more specific instructions, for example “take as few steps as possible,” may have increased conscious attention toward the coming perturbation. Second, there was only one day of practice and only 24 h to evaluate retention. Future studies should focus on longer compensatory step training programs and should investigate whether improvements over training can be retained over a longer period of time. Finally, the lack of improvement in performance in younger adults may have been due to a floor effect. This size was chosen because it elicited steps in both young and older adults, and did not result in falls in the older group. However, future studies should consider more challenging perturbations to elicit worse initial performance in healthy young adults, thus providing them room to improve.

In summary, this study showed that older adults possess an intact ability to improve compensatory stepping in response to repeated external perturbations of unknown direction and are able to retain this postural motor learning over 24 h. However, neither young nor older adults could generalize adaptations made during step training in the anteroposterior direction to lateral stepping. Future research should focus on more comprehensive, variable training programs that also involve lateral step training and determine whether such training could reduce fall incidence in daily life.

## Funding

This work was supported by the United States Department of Veteran's Affairs Rehabilitation Research and Development Service (Career Development Award-1: #I01BX007080; PI: DP) and VA Merit Award (E1075-R; PI: FH), the National Institutes of Health (R01 AG006457 29 PI: FH), and the Medical Research Foundation of Oregon (Early Investigator Award; PI: DP). The contents do not represent the views of the U.S. Department of Veterans Affairs or the United States Government.

### Conflict of interest statement

Dr. Horak has an equity/interest in APDM, a company that may have a commercial interest in the results of the study. This potential conflict of interest has been reviewed and managed by the Research and Development Committee at the Portland VA Medical Center. The authors declare that the research was conducted in the absence of any commercial or financial relationships that could be construed as a potential conflict of interest.
