# Supplementary material for: Older adults can improve compensatory stepping with repeated postural perturbations
Source: Front Aging Neurosci. 2015 Oct 21;7:201. doi: 10.3389/fnagi.2015.00201 (PMC4612504; doi:10.3389/fnagi.2015.00201)
Supplement: Supplementary file 1 [file DataSheet1.DOC]

**Supplementary material**

**Method: Step strategy in lateral stepping**

For medio-lateral perturbations, stepping strategy was manually identified. The following three lateral stepping strategies were identified: side-step, cross-over step and no step (Maki et al., 2000; Mille et al., 2005). The percent of trials using each strategy was calculated and were compared between Day 1 and Day 2.

**Results: Step strategy in lateral stepping**

The anterior-posterior compensatory step training did not change the percentage of steps used in lateral compensatory stepping between day 1 and day 2.

The percentage of cross over steps on day 1 did not change between young and older adults; U=63.5, p=.42 (two-tailed). The percentage of cross over steps did not change on day 2 for both older adults (z=-1.10, p=.34) and young adults (z=-1.53, p=.13). Collisions during cross over steps occurred in 2.54% of the trials on both days for the older adults. Collisions during cross over steps in young adults occurred in 3.08% of the trails on day 1 and 1.54% of the trials on day 2.

Older adults showed a significantly higher percentage of side steps on day 1 compared to young adults; U=38, p=<.05 (two-tailed). Older adults showed a trend for a lower percentage of side steps on day 2 (z=-1.78, p=.09), where young adults did not change (z=-0.95, p=.38).

Young adults showed a significantly higher percentage of no steps on day 1 compared to older adults; U=42, p=<.01 (two-tailed). Older adults always took steps on both days. Young adults did not change the percentage of no steps on day 2; z=-.63, p=.56
